# Regulatory mechanisms of *Nitraria tangutorum* in response to drought stress and functional verification of *NtPP2C51-like*

**DOI:** 10.3389/fpls.2026.1964307

**Published:** 2026-10-28

**Authors:** Xiaolan Li, Hanghang Liu, Yi Li

**Affiliations:** College of Forestry, Gansu Agricultural University, Lanzhou, China

**Keywords:** drought stress, *Nitraria tangutorum*, NtPP2C51-like, physiology and biochemistry, transcriptomics

## Abstract

*Nitraria tangutorum* is a pioneer shrub species for windbreak and sand fixation in desert regions. Drought stress caused by continuous global warming poses a significant environmental challenge to the growth and development of *N. tangutorum*. This study employed the *N. tangutorum* 2-17-16 from the Zhangye provenance as the experimental material to analyze the physiological and biochemical changes and transcriptome data of *N. tangutorum* seedlings under PEG-simulated drought stress, systematically elucidating the regulatory mechanisms underlying responses to drought stress in *N. tangutorum* at physiological and molecular levels. Furthermore, the function of the core regulatory gene *NtPP2C51-like* in drought resistance responses of *N. tangutorum* was investigated via heterologous overexpression in *Nicotiana benthamiana*. The results indicated that *N. tangutorum* responded to PEG-simulated drought stress by specifically increasing the content of osmoprotectants and activity of antioxidant enzymes. Under PEG-simulated drought stress, differentially expressed genes were significantly enriched in pathways such as Plant hormone signal transduction and MAPK signaling pathway-plant. Genes such as *IAA*, *GID1*, *ETR*, *EBF1/2*, and *PP2C* play significant roles in the response of *N. tangutorum* to drought stress. Under drought stress, *N. benthamiana* plants overexpressing *NtPP2C51-like* exhibited faster water loss, higher MDA content, lower contents of osmoprotectants, and lower antioxidant enzyme activities than wild-type plants. At 7 d of drought treatment, MDA content in the overexpression lines was 1.31–1.39-fold higher, whereas soluble sugar content was only 57–61% of that in wild-type plants, indicating that *NtPP2C51-like* overexpression increases drought sensitivity in the heterologous *N. benthamiana* system and suggesting a potential negative role of *NtPP2C51-like* in the drought response of *N. tangutorum*. These results provide a theoretical basis for understanding the physiological and molecular mechanisms underlying drought adaptation in *N. tangutorum* and provide candidate genes for further drought-tolerance studies and genetic improvement of this ecologically important desert plant overall.

## 1 Introduction

*Nitraria tangutorum* Bobrov, a perennial deciduous shrub belonging to the genus *Nitraria* of the family Nitrariaceae, is primarily distributed in lake basin sandy land, river terraces, mountain front plain sand accumulation land and clay land with wind-accumulated sands in deserts and desert grasslands, and is a pioneer shrub species for wind prevention and sand fixation in desert areas (Zhu et al., 2021). *N. tangutorum* is a xerophytic heliophyte that typically grows to a height of 0.5-2 meters, with branches that are prostrate or ascending. It has the characteristics of drought tolerance, heat tolerance, cold tolerance, salt-alkali tolerance, poor soil tolerance, and wind and sand resistance, making it a typical constructive species in desert and semi-desert areas (Zhou et al., 2015). The fruit of *N. tangutorum* is an ovate or oval berry-like drupe. Its hard endocarp effectively protects the seeds from harsh environments such as drought and high temperatures, ensuring germination when conditions are favorable (Hubert et al., 2024). Its branches sacrifice longitudinal extension to enhance lateral branches, creating a low and compact plant type to minimize the water consumption of new tissues and the overall transpiration surface area of the plant. Its leaves are fleshy and lanceolate, clustered in groups of 2-3 (or 4), covered with a waxy layer on the surface that effectively locks in moisture and reflects strong light (Liu et al., 2022). The branches and leaves of *N. tangutorum* are rich in osmotic adjustment substances such as proline and betaine, which can be rapidly accumulated during water stress to maintain cellular osmotic balance and prevent cell damage due to water loss (Li et al., 2024; Wei et al., 2024). Its root system is well developed, with many branches and strong sprouting ability, and it can produce a large number of adventitious roots rapidly after being buried in sand and soil, thus forming new plants and expanding the thicket to form fixed and semi-fixed *N. tangutorum* coppice dunes, which can trap and stabilize large amounts of quicksand, prevent the expansion and erosion of deserts, and play an important role in maintaining the balance of regional ecosystems and improving the quality of the ecological environments (Yin et al., 2020). The climate in desert areas is dry, precipitation is scarce and varies greatly in time and space, water resources are scarce, strong evaporation capacity, and the ecological environment is extremely fragile. Over the past century, persistent global warming have exacerbated the survival challenges of water shortage to desert flora, reduced the resilience and recovery capacity of desert ecosystems, and posed a long-term threat to ecological security in arid regions (Jia et al., 2016).

Water is a key factor for the survival of desert plants, which determines the survival, growth and spatial distribution of plants. Under the backdrop of global climate change, the limiting effect of water on desert vegetation is becoming increasingly pronounced, and vegetation restoration is becoming more and more difficult (Mu et al., 2024). Drought stress not only affects the morphological characteristics of plants, but also induces a reduction in the hydration of plant cells, an imbalance in osmotic pressure, accumulation of reactive oxygen species and metabolic disorders, seriously affecting the normal growth and development of plants (Zhou et al., 2021). Osmoregulation and antioxidant enzyme systems are crucial pathways for plants to cope with abiotic stress. Under drought stress, plants maintain cell turgor stability and water uptake capacity of plant cells by accumulating osmoregulatory substances such as proline, soluble sugars, and soluble proteins (Luo and Yuan, 2019). In addition, plants can induce a series of changes in antioxidant enzyme activities to eliminate excessive accumulation of reactive oxygen species in cells, reduce the extent of membrane lipid peroxidation, and decrease malondialdehyde (MDA) content, thereby alleviating the oxidative damage caused by drought stress to plant cells (Niu et al., 2025; Yang et al., 2021). In exploring the physiological and biochemical responses of peanut to drought stress, Sun et al. demonstrated that prolonged drought stress triggers a burst of reactive oxygen species, leading to severe membrane lipid peroxidation and a significant increase in MDA content (Sun et al., 2024). They believed that peanuts enhance cell water retention and stress tolerance capacity through a synergistic increase in the activity of antioxidant enzymes such as superoxide dismutase (SOD) and peroxidase (POD), as well as the accumulation of osmotic adjustment substances including soluble proteins, soluble sugars, and proline. The physiological and biochemical responses of plants to drought stress are not merely passive responses, but rather active adaptations precisely regulated by a complex molecular signal transduction network (Hosseini et al., 2025). Under drought stress, plants sense external stress signals through specific receptors, transmit them via hormones and second messengers, activate the transcription factor network, recognize and bind to specific sequences in the promoter region of target genes, regulate the expression of functional proteins, and drive physiological and biochemical responses (Ali et al., 2025). High-throughput sequencing technology is a powerful tool for analyzing the transcriptional regulatory network, transcriptional expression dynamics and differential expression characteristics of plant genomes under various environmental conditions or physiological states. Zhi et al. conducted transcriptome analysis on *Leucaena* leaves under PEG-simulated drought stress and found that the enrichment degree of differentially expressed genes (DEGs) in the plant hormone signal transduction pathway was the highest, and genes related to ABA biosynthesis and signal transduction (*NCED*, *PP2C*, *ABF*) were specifically induced and upregulated (Zhi et al., 2024).

Protein Phosphatases (PPs) are core components of the complex signal transduction network in plants and play a crucial role in regulating plant growth and development as well as responses to abiotic stress (Shazadee et al., 2019). The Protein Phosphatase 2C (*PP2C*) family is the largest branch of plant protein phosphatases, with its members classified into 12 subfamilies (A-L) and 6 independent clades in phylogenetic taxonomy (Fuchs et al., 2013). Subgroup A *PP2Cs*, as key negative regulators in the abscisic acid (ABA) signaling pathway, together with ABA receptors Pyrabactin Resistance 1 (PYR1)/PYR1-like (PYL)/Regulatory Components of ABA Receptors (RCAR) and sucrose non-fermenting 1-related protein kinase 2 (SnRK2s), constitute the three main regulatory components of the ABA signaling pathway (Shen et al., 2022; Yang et al., 2025). Under normal conditions, *PP2Cs* of subgroup A interact with SnRK2s to dephosphorylate SnRK2s and reduce its kinase activity, thereby inhibiting ABA response. When stimulated by developmental or environmental signals, ABA receptors PYR1/PYL/RCAR interact with A subgroup *PP2Cs* after binding to ABA molecules, releasing SnRK2s from the inhibition of *PP2Cs*. SnRK2s achieve self-activation through autophosphorylation and initiate downstream ABA responses (Lim et al., 2023; Wang et al., 2023). The ABA-PYR/PYL/RCAR-PP2C-SnRK2 signaling transduction pathway, as the core regulatory hub for plant perception and response to drought stress, plays an important role in the cascade response of plant drought resistance (Riaz et al., 2025). Zeng et al. cloned and functionally identified the *PP2C26* gene of cassava A subfamily in exploring the regulatory mechanism of cassava in response to abiotic stress. The results showed that the transcription level of *MePP2C26* was upregulated induced by PEG, NaCl, and ABA. Compared with wild-type *Arabidopsis thaliana*, overexpressed *MePP2C26 Arabidopsis thaliana* had increased drought sensitivity. It is believed that the *MePP2C26* gene negatively regulates the response of cassava to drought stress (Zeng et al., 2025). Based on these findings, we hypothesized that subfamily A PP2Cs may play a negative regulatory role in the drought response of *N. tangutorum*. This study used the desert plant *N. tangutorum* as the experimental material. The regulatory mechanism of *N. tangutorum* in response to drought stress was systematically illustrated from the physiological and molecular levels by analyzing the physiological and biochemical changes and transcriptome data of *N. tangutorum* seedlings under PEG-simulated drought stress. Furthermore, functional verification of the core regulatory gene *NtPP2C51-like* was conducted through heterologous overexpression in *Nicotiana benthamiana* to elucidate the key pathways by which *N. tangutorum* responds to drought stress. This study aimed to establish a theoretical basis for understanding physiological and molecular mechanisms underlying drought adaptation in *N. tangutorum* and identify candidate genes for further drought-tolerance studies and genetic improvement of this ecologically important desert plant.

## 2 Materials and methods

### 2.1 Experiment materials and treatments

Drought-tolerant elite *N. tangutorum* seedlings from a selected provenance in Zhangye, designated 2-17-16, were used as the experimental materials. The seeds were obtained from the Gansu Province Academy of Qilian Water Resource Conservation Forests Research Institute (Zhangye, China). On 15 April 2025, the intact plump seeds were disinfected with 20% sodium hypochlorite solution for 15 min and washed with distilled water before being planted in pots filled with nutrient soil. They were placed in a plastic greenhouse at Gansu Agricultural University for standard greenhouse management. In early July 2025, uniformly growing *N. tangutorum* seedlings were selected and transplanted individually into independent hydroponic containers containing 1/2 Hoagland nutrient solution, where they were maintained under day/night temperatures of 26 ± 2 °C/20 ± 2 °C, natural sunlight with a natural photoperiod, and relative humidity of 45–60%. Based on previous studies (Wei et al., 2024) and the results of preliminary experiments, the seedlings were subjected to drought stress simulated by treatment with 1/2 Hoagland nutrient solution containing 20% PEG-6000 after a 7-day acclimation period. At 0, 6, 12, and 24 h after PEG-6000 treatment, seven seedlings were randomly selected from the PEG-treated population and randomly allocated into three non-overlapping groups, with two or three seedlings per group. The root, stem, and leaf tissues from the seedlings within each group were collected separately and pooled to generate one pooled sample for each tissue type. Thus, three independent pooled biological replicates were obtained for each tissue type at each sampling time point. The removed samples were immediately placed into liquid nitrogen for quick freezing and stored at −80 °C for physiological index determination and RNA extraction. The sterile seedlings of *N. benthamiana* used for genetic transformation in this study were subcultured and preserved in our laboratory. *Escherichia coli* DH5α and *Agrobacterium tumefaciens* GV3101 competent cells were purchased from Beijing Solarbio Science & Technology Co., Ltd., (Beijing, China).

### 2.2 Physiological parameters determination

The physiological and biochemical parameters of roots, stems and leaves of *N. tangutorum* under different time of PEG-simulated drought stress were determined to elucidate the physiological response mechanisms of different tissues of *N. tangutorum* to drought stress. The MDA content was measured by the thiobarbituric acid (TBA) reaction (Sebastiano et al., 2025). Soluble sugar content was calculated in mg/g FW by a modified method of Ichsan et al (Ichsan et al., 2022). Soluble protein concentrations were measured using the Coomassie Brilliant Blue G-250 staining method of Bradford (Bradford, 1976). Proline concentration was measured by the method of Bates (Bates et al., 1973). Starch content was calculated according to the method described by Chow and Landhausser (Chow and Landhäusser, 2004). Peroxidase (POD), superoxide dismutase (SOD), and catalase (CAT) activities were quantified using reagent kits (Beijing Solarbio Science & Technology Co., Ltd., Beijing, China) following the manufacturer’s protocol. Three biological replicates were taken from each sample. Data were analyzed using one-way analysis of variance (ANOVA), followed by Duncan’s multiple range test in IBM SPSS Statistics 26.0. Statistical significance was set at P < 0.05. Data are presented as means ± SD.

### 2.3 Transcriptomic analysis

#### 2.3.1 Library construction and illumina sequencing

Total RNA of the samples was extracted using a plant RNA extraction kit (Tiangen, Beijing, China) in accordance with the manufacturer’s instructions. RNA integrity was verified by 1% agarose gel electrophoresis, and RNA concentration and purity were assessed using a NanoDrop 2000 spectrophotometer (Thermo Fisher Scientific, Waltham, MA, USA). The obtained high-quality RNA (OD_260_/OD_280_ = 1.85-2.20, concentration ≥ 30 ng/μL) was finally used for library construction. The mRNA was isolated and enriched from the total RNA using poly-T oligo-attached magnetic beads. The target libraries were generated by reverse transcription of mRNA into cDNA followed by adapter ligation. After the library passed the quality inspection, it was sequenced on an Illumina NovaSeq 6,000 platform (Illumina, San Diego, CA, USA). Library construction and Illumina sequencing were performed with the help of Biomarker Technologies Co., Ltd. (Beijing, China). Due to the lack of a publicly available reference genome for *N. tangutorum*, the non-redundant full-length transcript sequences generated by PacBio SMRT sequencing in our research group were used as the custom reference transcriptome for the RNA-seq analysis.

#### 2.3.2 Identification and enrichment analysis of DEGs

The raw sequencing data were firstly processed by Fastp (https://github.com/OpenGene/fastp) software (Chen et al., 2018). In this step, clean reads were obtained by removing adapter sequences, reads containing more than 10% unknown bases and low-quality sequences from raw data. The quality of the clean reads was assessed based on Q20, Q30, GC-content, and sequence duplication levels. The clean reads were aligned to the custom reference transcriptome using STAR (v2.5.0b) with default parameters. Transcript abundance was quantified using Kallisto (v0.46.1). FPKM (Fragments Per Kilobase of transcript per Million fragments mapped) was used to evaluate the expression levels of genes or transcripts. Principal component analysis (PCA) was performed based on the FPKM expression matrix to assess the overall transcriptional relationships and reproducibility among the nine RNA-seq samples. DESeq2 (1.30.1) was utilized for gene differential expression analysis among samples (Love et al., 2014). Raw integer count data, rather than FPKM values, were used as input for DESeq2. DESeq2 was run with its default statistical settings (Wald test and parametric dispersion estimation). The default size-factor normalization implemented in DESeq2 was used to account for differences in sequencing depth among samples. Multiple testing was controlled using the Benjamini-Hochberg procedure, and DEGs were identified using a false discovery rate (FDR) < 0.01 and an absolute fold change (FC) ≥ 2. All the obtained DEG sequences were subjected to Gene Ontology (GO) and Kyoto Encyclopedia of Genes and Genomes (KEGG) enrichment analysis using topGO and KOBAS (2.0) R package, respectively, to identify the functions and metabolic pathways involved in DEGs (Alexa and Rahnenführer, 2010; Xie et al., 2011). The expression profiles of selected key drought-responsive genes were visualized as a heatmap based on FPKM values using BMKCloud (www.biocloud.net). In MEGA 12.1, the conserved PP2C domain sequences of the differentially expressed *PP2C* genes were subjected to multiple sequence alignment using ClustalW, followed by phylogenetic analysis using the Neighbor-Joining (NJ) method with 1,000 bootstrap replicates. Bootstrap values were displayed at the major internal nodes.

#### 2.3.3 Verification of RNA-Seq data by real-time quantitative PCR

To validate the reliability of the transcriptome data, qRT-PCR analysis was conducted on the expression level of 12 selected DEGs. The total RNA extraction method was the same as that used during library construction. A total of 1 µg of RNA was used for the reverse transcription reaction in accordance with the manufacturer’s instructions of the Prime Script TM RT Reagent Kit (TaKaRa, Dalian, China). The qRT-PCR was performed using ChamQ Universal SYBR qPCR Master Mix Kit (Vazyme, China) in Roche LightCycler^®^96 Detection System (Applied Biosystems, Foster City, CA, USA). The relative expression levels of the selected genes were normalized to the reference gene *EF1-α* (*EF1-α-*F: ACGCCTTGCTTGCTTTCAC; *EF1-α-*R: GATTTCATCGTACCTTGCCTTT). The reaction conditions were as follows: 95 °C for 30 s, followed by 40 cycles of 95 °C for 10 s, and 60 °C for 30 s. The relative expression of candidate genes was calculated according to the 2^−ΔΔ^*^C^*^t^ method and analyzed after log_2_ transformation. All gene-specific primers used in qRT-PCR were designed using Primer 3.0 online software and are listed in **Supplementary Table S1**.

### 2.4 Cloning and functional verification of *NtPP2C51-like*

#### 2.4.1 Construction of *NtPP2C51-like* overexpression vector

The CDS sequence of *NtPP2C51-like* was retrieved from the transcriptome data and specific primers were designed using Primer 3.0 online software (**Supplementary Table S1**). RNA was isolated from *N. tangutorum* seedlings and reverse transcribed into cDNA. PCR amplification of the target gene was performed using 3 µL of cDNA template in a total reaction volume of 40 µL. PCR amplification was performed using high concentration cDNA as template for the target gene. Reaction procedure: pre-denaturation at 95°C for 10 min; 95°C denaturation 30 s, 56°C annealing 30 s, extension at 72 °C for 60 s, 30 cycles; extension at 72 °C for 10 min. The amplified fragments were verified by 1% agarose gel electrophoresis and purified according to the instructions of the DNA Gel Extraction & DNA Purification Kit (Simgen, Hangzhou, China). Then, the purified product was ligated into the *pART-CAM-FLAG* vector and transformed into competent *E. coli* cells (DH5α strain) for sequencing. The plasmid of the sequenced successfully positive colony was extracted and transformed into *Agrobacterium tumefaciens* GV3101 to obtain the *Agrobacterium* engineered strain.

#### 2.4.2 Bioinformatics analysis of *NtPP2C51-like*

Sequence analysis of *NtPP2C51-like* was conducted using different bioinformatics tools to predict its function. The sequence similarity and protein domains of *NtPP2C51-like* were analyzed using NCBI BLAST (https://blast.ncbi.nlm.nih.gov/Blast.cgi) and NCBI CDD (https://www.ncbi.nlm.nih.gov/cdd/). The homologous sequences of NtPP2C51-like proteins were retrieved from the public protein database of NCBI, and the phylogenetic tree was constructed using the Neighbor-Joining (NJ) method in MEGA 12.1 software.

#### 2.4.3 Generation of transgenic *N. benthamiana* and functional verification of *NtPP2C51-like*

The 6-week-old *N. benthamiana* tissue-cultured plantlets with uniform growth were selected for *Agrobacterium*-mediated genetic transformation (Ni et al., 2025). In the laminar flow hood, leaves of tissue-cultured plantlets were cut into 1 cm^2^ pieces and immersed in a pre-prepared *Agrobacterium* suspension with an OD_600_ of 0.4 to infect for 10 min. After infection, the leaves were blotted dry with sterile filter paper, placed adaxial side up on MS medium and cultured at 25°C in darkness for 2-4 d. Subsequently, they were transferred into regeneration medium containing 100 mg/L kanamycin and 250 mg/L cephalosporin to induce shoot formation. When the regenerated buds grow to 2 cm, transfer them to a rooting medium for further cultivation. Select resistant regenerated plants with strong root systems, extract total DNA from leaves, and conduct positive identification by PCR to obtain transgenic plants. Wild-type and transgenic *N. benthamiana* plants with uniform growth were transplanted into flowerpots filled with the same quality of nutrient soil and placed in light incubators for adaptive cultivation. Culture conditions: temperature 25 ± 1°C, light intensity 2000 lx, 16 h light/8 h dark, and relative humidity 60%-70%. After one week of acclimation, 16 seedlings were randomly selected for each line, including the wild-type (WT) and three transgenic lines (OE1, OE2, and OE3). All selected plants were then watered to saturation, allowed to drain for 2 h, and subsequently subjected to natural drought stress. At each time point (days 0, 3, 5, and 7), three seedlings from each line were randomly selected for phenotypic observation, followed by sampling. Phenotypic observations were conducted at 9:00–10:00 AM on each sampling day, after which the third to sixth leaves were collected for determination of the expression level of *NtPP2C51-like* and physiological and biochemical parameters. Each individual seedling was considered one biological replicate (n = 3). The expression level of *NtPP2C51-like* was determined by qRT-PCR, with *EF1-α* used as the reference gene for normalization. Relative transcript levels were calculated using the 2^−ΔΔ^*^C^*^t^ method. Data were analyzed using one-way analysis of variance (ANOVA), followed by Duncan’s multiple range test in IBM SPSS Statistics 26.0. Statistical significance was set at P < 0.05. Data are presented as means ± SD.

## 3 Results

### 3.1 Effects of PEG-simulated drought stress at different times on MDA and osmoprotectant contents in different tissues of *N. tangutorum* seedlings

MDA is an important indicator for assessing the degree of cell membrane damage and membrane lipid peroxidation in plants under adverse stress. As shown in **Figure 1A**, the MDA content in roots, stems and leaves of *N. tangutorum* seedlings continuously increased with the prolongation of PEG-simulated drought stress. After 24 h of PEG-simulated drought stress, the MDA content in roots, stems and leaves was 2.04, 1.37, and 1.28 times that of the control, respectively. Osmoregulatory substances such as soluble sugar, soluble protein, and proline are important strategies for plants to resist adverse stress and alleviate drought damage. As shown in **Figure 1B**, the soluble sugar content in roots, stems and leaves of *N. tangutorum* seedlings gradually increased with the extension of PEG-simulated drought stress time. Compared with the control, the soluble sugar content in the stem did not increase significantly after 6 h of PEG-simulated drought stress, but reached 1.69- and 1.91-fold that of the control after 12 and 24 h, respectively. The soluble sugar content in roots and leaves was significantly increased under PEG-simulated drought stress at different time points compared with the control. After 24 h stress, the soluble sugar content in roots and leaves was 2.36 and 1.38 times that of the control, respectively. As shown in **Figure 1C**, the soluble protein content in roots, stems, and leaves of *N. tangutorum* seedlings showed an upward trend with the prolongation of PEG-simulated drought stress time. After 24 h stress, the soluble protein contents in roots, stems and leaves were 1.90, 2.30, and 2.78 times that of the control, respectively. As shown in **Figure 1D**, the proline content in roots, stems, and leaves of *N. tangutorum* seedlings increased significantly with the extension of PEG-simulated drought stress time, reaching 1.75-, 1.69-, and 1.50-fold that of the control at 24 h, respectively.

**Figure 1:**
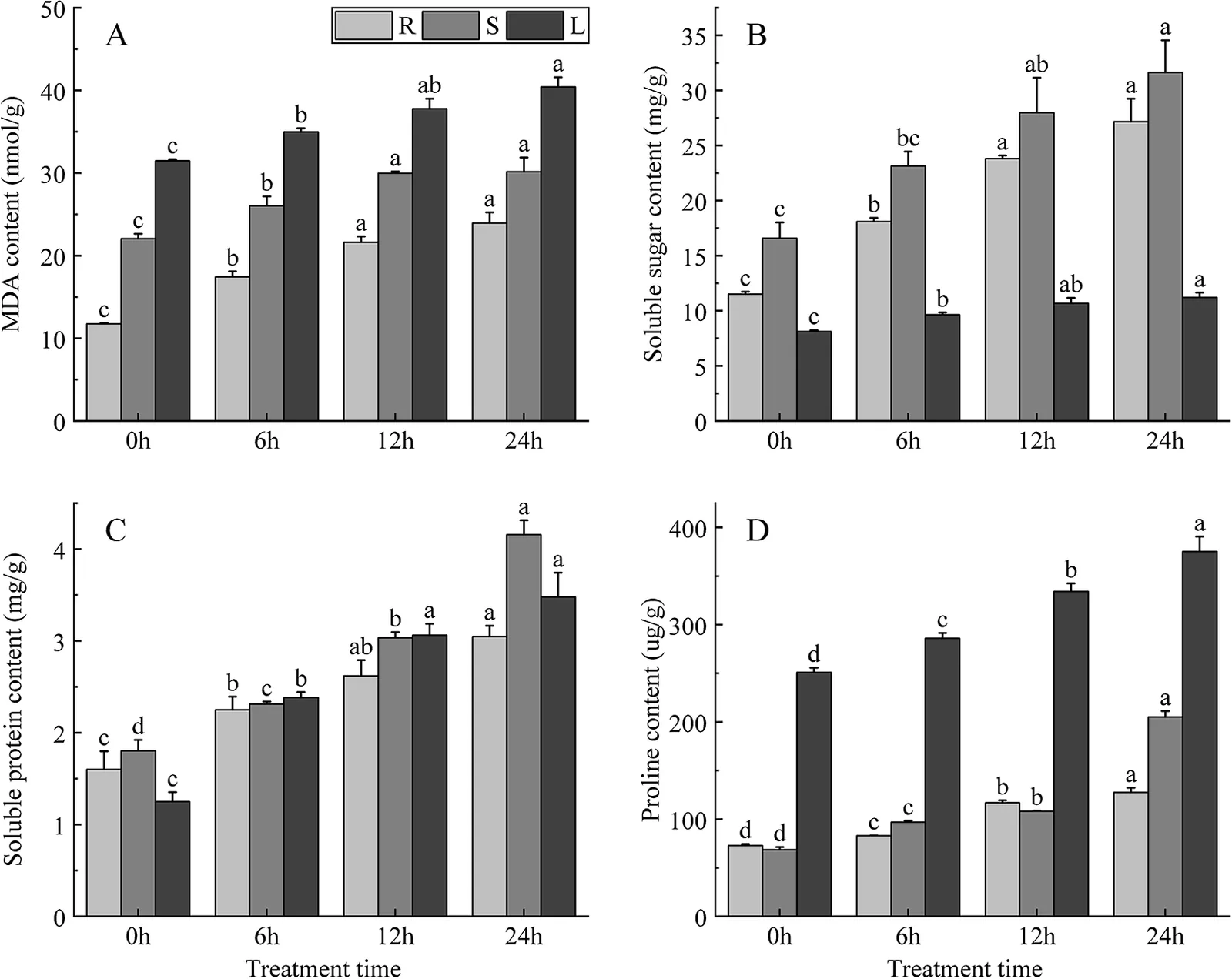
Effects of PEG-simulated drought stress at different times on MDA and osmoprotectant contents in different tissues of *N. tangutorum* seedlings. **(A)** MDA content; **(B)** Soluble sugar content; **(C)** Soluble protein content; **(D)** Proline content. 0 h, 6 h, 12 h, and 24 h denote the different time points sampled during PEG-simulated drought stress. Different lowercase letters above the error bars indicate statistically significant differences among different drought stress time points within the same tissue (ANOVA, *p* < 0.05). Data are presented as mean ± SE (n = 3 biological replicates).

### 3.2 Effects of PEG-simulated drought stress at different times on starch content and antioxidant enzyme activities in different tissues of *N. tangutorum* seedlings

Starch plays a crucial role in plant response to abiotic stress and maintaining the stability of cell functions. As shown in **Figure 2A**, the starch content in roots, stems and leaves of *N. tangutorum* seedlings decreased gradually with the prolongation of PEG-simulated drought stress time. At 6 h of PEG-simulated drought stress, the starch content in leaves did not decrease significantly compared with the control, but decreased significantly by 26.97% and 43.33% after 12 and 24 h of stress, respectively. The starch content in roots and stems was significantly decreased under PEG-simulated drought stress at different time points compared with the control, with reductions of 56.09% and 53.25% at 24 h, respectively. POD, SOD, and CAT jointly maintain the balance of reactive oxygen species metabolism in plant cells and alleviate oxidative damage caused by drought. As shown in **Figure 2B**, the POD activity in roots, stems, and leaves of *N. tangutorum* seedlings was significantly increased under PEG-simulated drought stress at different time points compared with the control, reaching 2.75-, 1.66-, and 1.53-fold that of the control at 24 h, respectively. As shown in **Figure 2C**, the SOD activity in roots, stems, and leaves of *N. tangutorum* seedlings showed an upward trend with the extension of stress time. The SOD activity in leaves did not increase significantly at 6 h of PEG-simulated drought stress compared with the control, and that in roots and stems increased significantly at all stress time points. After 24 h of PEG-simulated drought stress, the SOD activity in the roots, stems, and leaves was 2.28, 1.42, and 1.47 times that of the control, respectively. As shown in **Figure 2D**, the CAT activity in stems and leaves of *N. tangutorum* seedlings increased significantly with the prolongation of PEG-simulated drought stress time. The CAT activity in roots did not increase significantly at 12 h of PEG-simulated drought stress compared with that at 6 h. After 24 h of PEG-simulated drought stress, CAT activity in the roots, stems, and leaves was 2.50-, 1.80-, and 2.01-fold that of the control, respectively.

**Figure 2:**
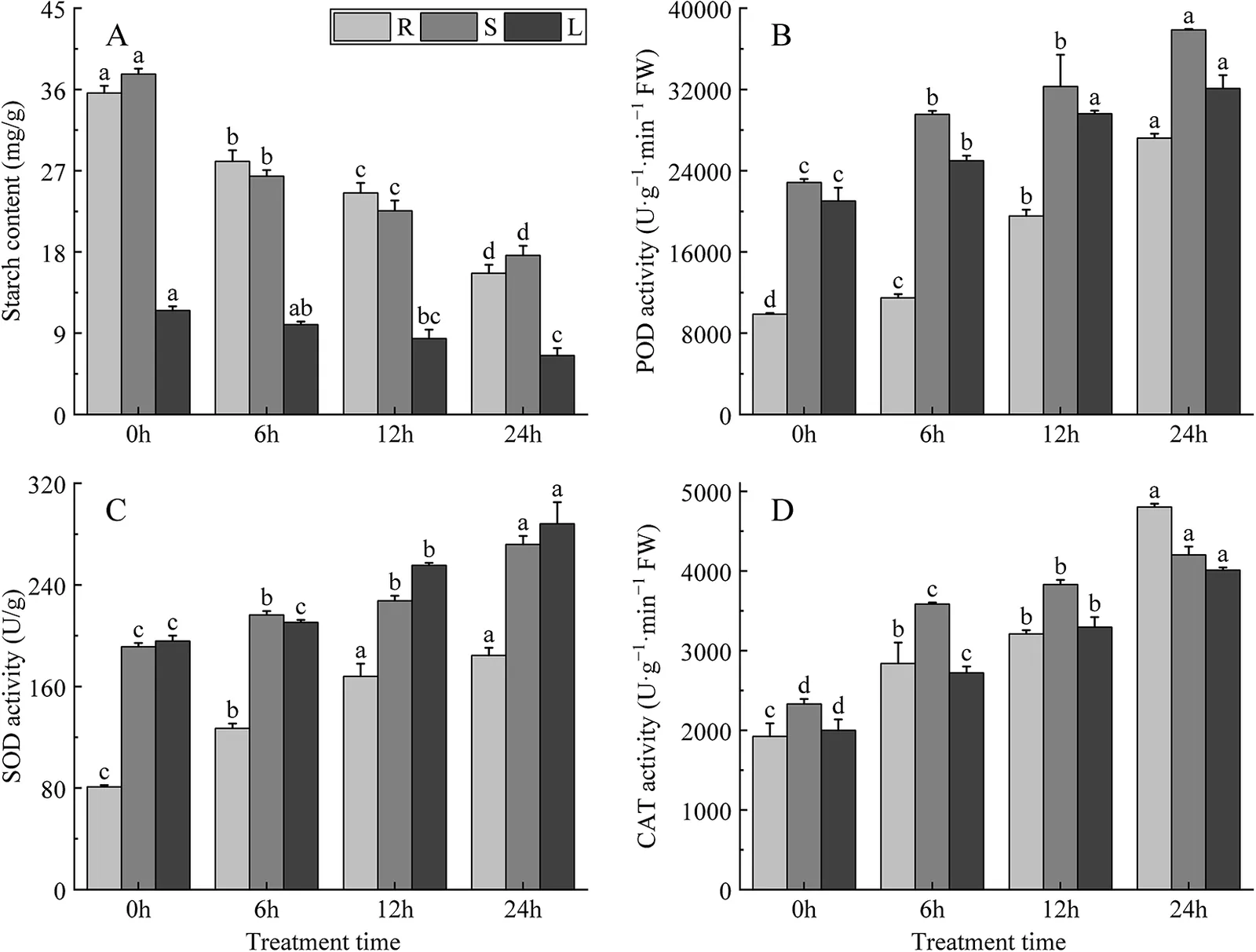
Effects of PEG-simulated drought stress at different times on starch content and antioxidant enzyme activities in different tissue of *N. tangutorum* seedlings. **(A)** Starch content; **(B)** POD activity; **(C)** SOD activity; **(D)** CAT activity. 0 h, 6 h, 12 h, and 24 h denote the different time points sampled during PEG-simulated drought stress. Different lowercase letters above the error bars indicate statistically significant differences among different drought stress time points within the same tissue (ANOVA, *p* < 0.05). Data are presented as mean ± SE (n = 3 biological replicates).

### 3.3 Transcriptome sequencing

Based on the changing trends and response characteristics of physiological and biochemical indicators in different tissues of *N. tangutorum* seedlings under PEG-induced drought stress at different time points, root tissue was selected for transcriptome sequencing. At 24 h of PEG treatment, roots showed the greatest relative changes in 7 of the 8 physiological and biochemical indicators examined, including MDA, soluble sugar, proline, starch, POD, SOD, and CAT, indicating a relatively pronounced and consistent response to PEG-induced osmotic stress. In addition, roots were directly exposed to the PEG-containing solution and therefore represented the tissue most directly subjected to the imposed osmotic stress. Accordingly, root samples collected at 0 h, 6 h, and 24 h were used for transcriptome sequencing. Each sample conducted three biological replicates to explore the molecular response mechanism of *N. tangutorum* to drought stress. After removing low-quality and redundant sequences, a total of 61.78 Gb of clean reads were obtained from 9 cDNA libraries, with each library generating at least 6.28 Gb of clean reads. The base coverage rates of Q20 and Q30 in all libraries were above 97.73% and 93.95%, respectively, and the GC content ranged from 44.78% to 45.28%. The clean reads were aligned to the custom reference transcriptome using STAR, and the mapping rates of the nine libraries ranged from 79.63% to 82.40% (**Figure 3A**). These results indicated that the sequencing data were of high quality and suitable for subsequent transcriptomic analyses.

**Figure 3:**
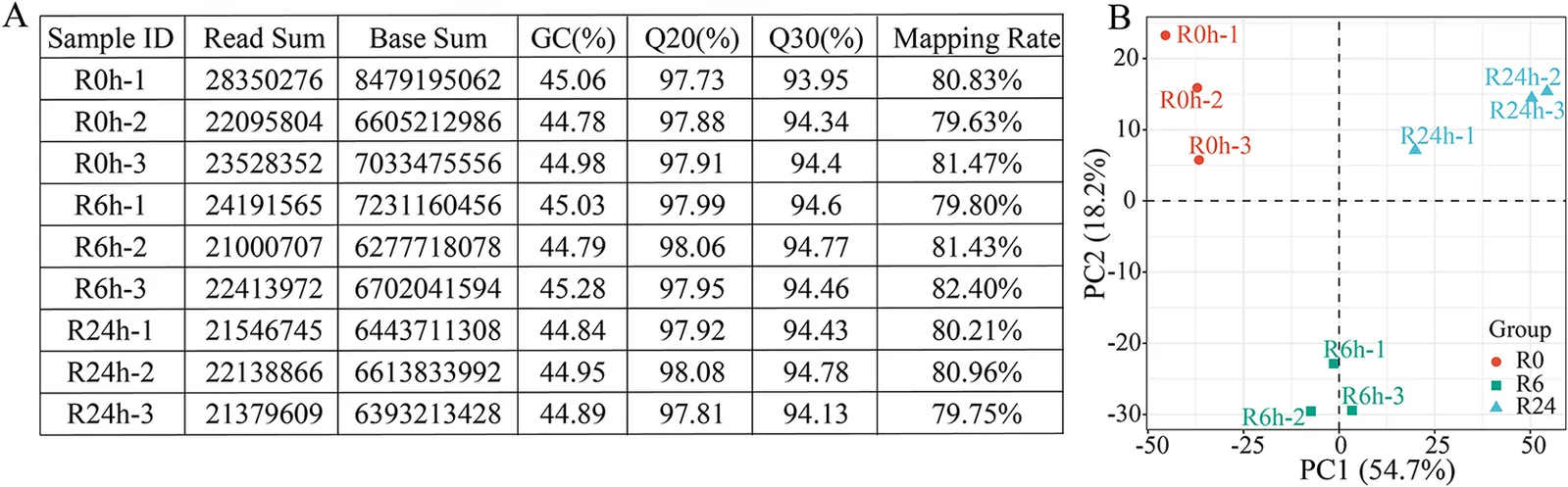
Sequencing quality, mapping statistics, and principal component analysis of root RNA-seq samples. **(A)** Sequencing quality and mapping statistics of nine RNA-seq libraries, including read number, base number, GC content, Q20, Q30, and mapping rate. **(B)** PCA based on the gene expression matrix of the nine root samples. R0h, R6h, and R24h indicate sampling at 0, 6, and 24 h after PEG-6000 treatment, respectively. Numbers 1–3 indicate biological replicates.

Principal component analysis (PCA) based on the FPKM expression matrix showed clear separation among the three sampling time points, while the three biological replicates within each time point clustered relatively closely together (**Figure 3B**). PC1 and PC2 explained 54.7% and 18.2% of the total variation, respectively, accounting for 72.9% of the cumulative variation. The relatively close clustering of biological replicates indicated good reproducibility among the samples and supported the reliability of the transcriptome dataset for subsequent differential expression and functional enrichment analyses.

### 3.4 Identification and enrichment analysis of DEGs

Using Fold Change ≥ 2 and FDR < 0.01 as the criteria for identifying DEGs, a total of 2,455 DEGs were identified in the three comparison combinations of this study. Compared with the control, 259 genes were significantly upregulated and 189 genes were significantly downregulated after 6 h of PEG-simulated drought stress (R0h vs R6h) (**Figure 4A**). A total of 1,170 upregulated genes and 777 downregulated genes were detected after 24 h of PEG-simulated drought stress compared with the control (R0h vs R24h). The number of differentially expressed genes was the highest at 24 h of PEG-simulated drought stress, with 301 genes significantly upregulated and 318 genes significantly downregulated compared with 6 h of stress (R6h vs R24h). In addition, nine commonly DEGs were identified across the three comparison groups, indicating that these genes play a crucial role in the response of *N. tangutorum* to drought stress (**Figure 4B**).

**Figure 4:**
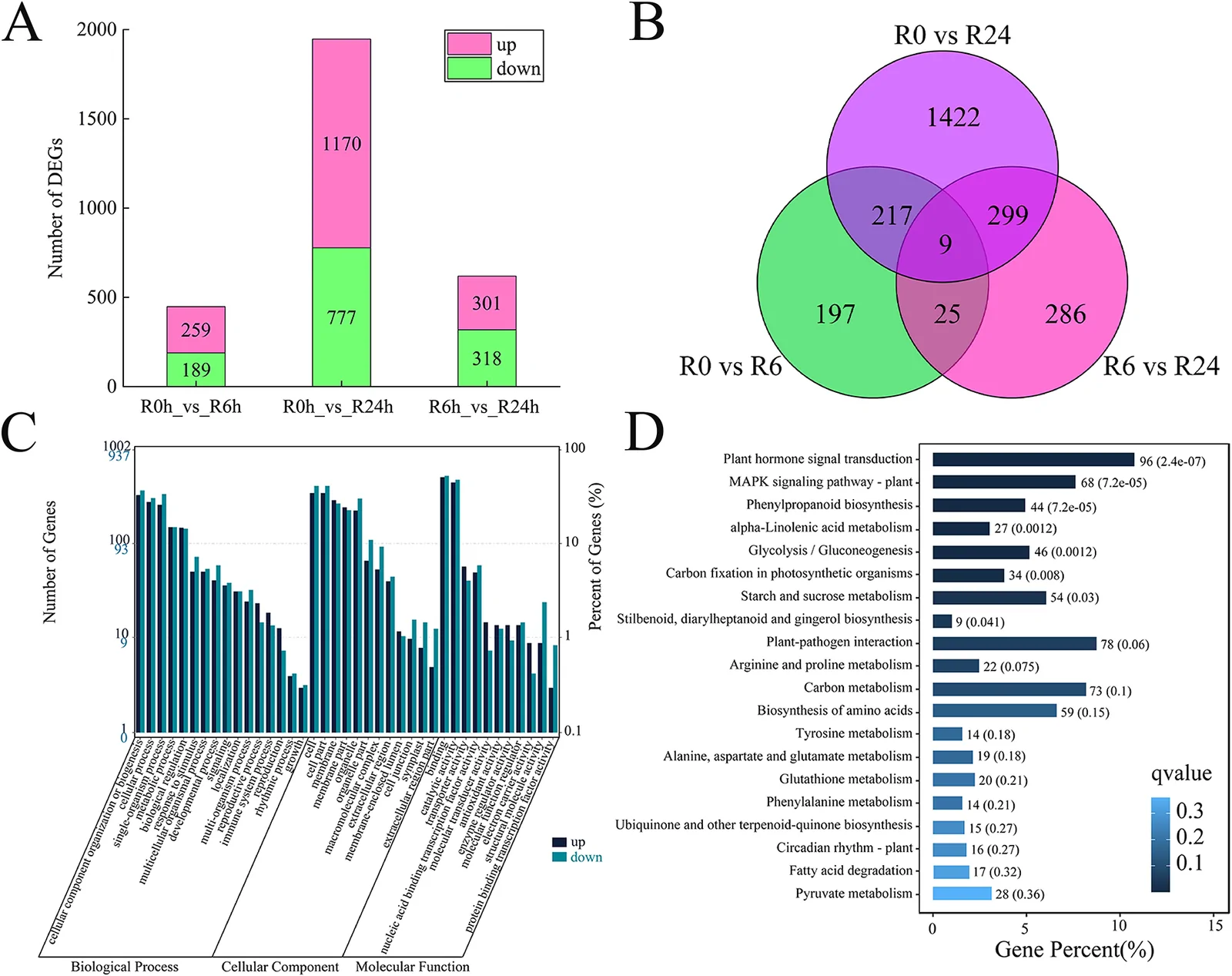
Statistics and enrichment analysis of DEGs. **(A)** Statistics on the number of DEGs; **(B)** Venn diagram analysis of expressed genes between differential groups; **(C)** GO analysis of DEGs; **(D)** KEGG pathway enrichment analysis of DEGs.

Functional annotation and classification of all drought-responsive DEGs in *N. tangutorum* seedlings were performed using GO enrichment analysis (**Figure 4C**). The 2,455 DEGs were primarily enriched in 39 GO terms, comprising 16 biological process (BP) terms, 12 cellular component (CC) terms, and 11 molecular function (MF) terms. In the BP classification, GO terms such as cellular component organization or biogenesis (GO:0071840), cellular process (GO:0009987), single-organism process (GO:0044699), metabolic process (GO:0008152), and biological regulation (GO:0065007) were significantly enriched. The five most significantly enriched GO terms in CC are Cell (GO:0005623), cell part (GO:0044464), membrane (GO:0016020), membrane part (GO:0044425), and organelle (GO:0043226). Binding (GO:0005488) and catalytic activity (GO:0003824) were significantly enriched in the MF.

The biological functions of all drought-responsive DEGs were further analyzed using the KEGG database, and the top 20 significantly enriched metabolic pathways were visualized. As shown in **Figure 4D**, DEGs were significantly enriched in metabolic pathways such as Plant hormone signal transduction (ko04075), MAPK signaling pathway-plant (ko04016), Phenylpropanoid biosynthesis (ko00940), alpha-Linolenic acid metabolism (ko00592), Glycolysis/Gluconeogenesis (ko00010), Carbon fixation in photosynthetic organisms (ko00710) and Starch and sucrose metabolism (ko00500). The results indicate that signal transduction pathways play a crucial role in the response of *N. tangutorum* to drought stress.

### 3.5 Plant hormone signal transduction pathways and drought resistance of *N. tangutorum*

The plant hormone signal transduction pathway is the core regulatory system for plants to perceive adverse conditions, transmit stress signals, and coordinate growth and stress resistance. As shown in **Figure 5A**, signaling pathways such as auxin, cytokinin, gibberellin, abscisic acid, ethylene, and brassinolide activate the expression of transcription factors and downstream functional genes through specific receptor recognition and signal cascade transmission, and participate in the response of *N. tangutorum* to drought stress. In this study, a total of 78 DEGs were identified to be significantly enriched in the plant hormone signal transduction pathway, which together constitute a complex and precise drought response regulatory network (**Figure 5B**). Among them, the expression levels of Transcript68026 encoding *IAA* in the auxin signaling pathway, Transcript47327 encoding *GID1* in the gibberellin signaling pathway, as well as Transcript64555, Transcript1874, Transcript524, and Transcript26971 encoding *ETR* and *EBF1/2*, respectively, in the ethylene signaling pathway were significantly up-regulated with the prolongation of drought stress time. The results indicate that *IAA*, *GID1*, *ETR*, and *EBF1/2* genes play a significant role in the response of *N. tangutorum* to drought stress. The ABA signaling pathway, consisting of three core components ABA receptors PYR/PYL/RCAR, PP2Cs, and SnRK2s, is the primary pathway for plants to perceive and resist drought stress. In this study, one down-regulated *PYL* gene and eight up-regulated *PP2C* genes were screened in the ABA signaling pathway. Among them, Transcript32471 encoding *PP2C* was the most significantly up-regulated, suggesting that Transcript32471 may be involved in the ABA signaling pathway-mediated drought response of *N. tangutorum*.

**Figure 5:**
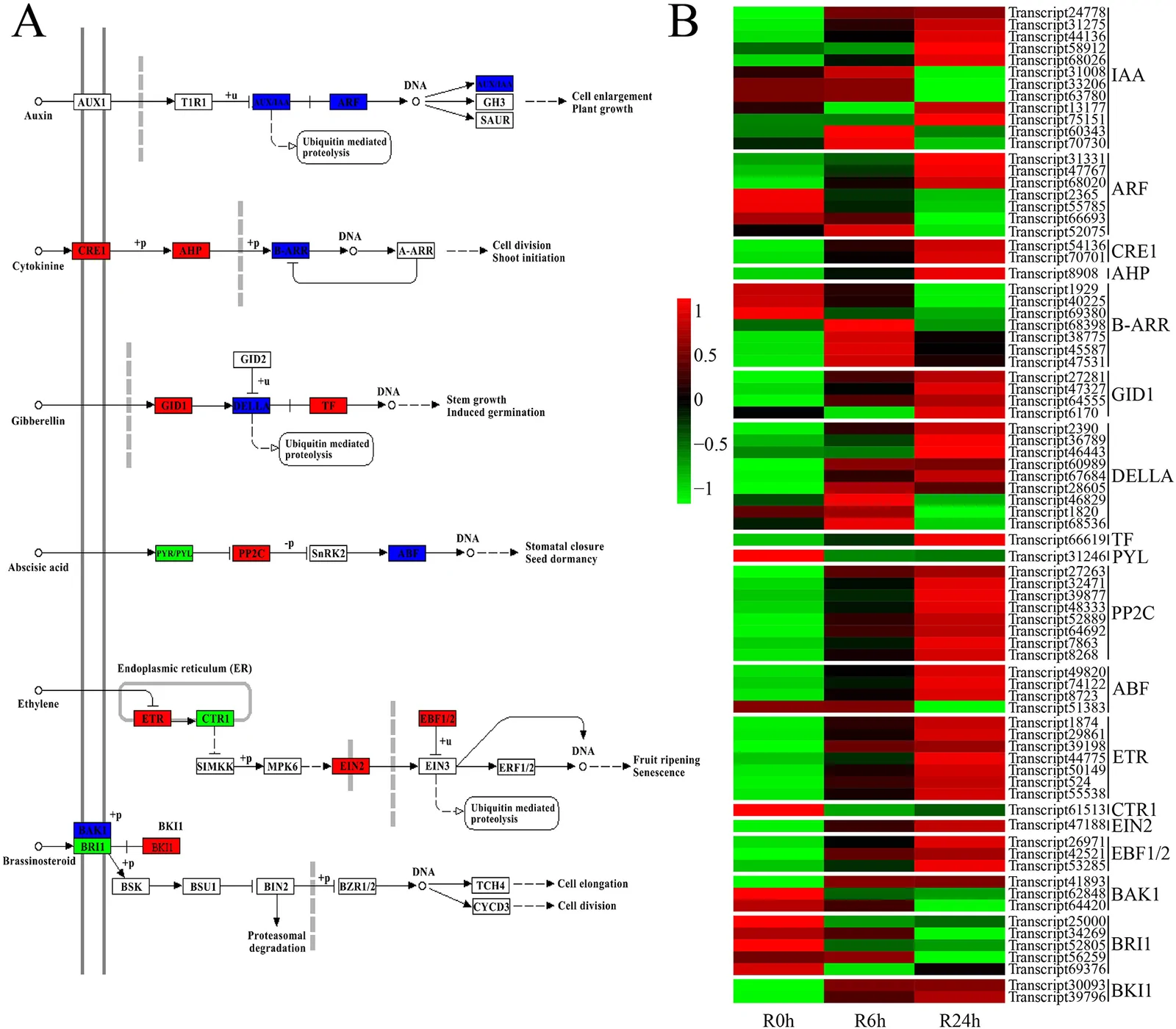
Plant hormone signal transduction pathways and heatmap analysis of DEGs annotated in these pathways. **(A)** Plant hormone signal transduction pathways; **(B)** Heatmap analysis of DEGs annotated in plant hormone signal transduction pathways.

### 3.6 Phylogenetic analysis of *NtPP2C* family member

The *PP2C* gene family plays an indispensable role in regulating plant growth and development, as well as stress response. In order to explore the role of *PP2C* family members in the response of *N. tangutorum* to drought stress, we screened 23 differentially expressed *PP2C* genes from the transcriptome-wide DEG dataset and conducted a phylogenetic analysis of these genes. Based on the phylogenetic relationships between the conserved PP2C domains of the *N. tangutorum* proteins and the corresponding *Arabidopsis thaliana* PP2C proteins, 23 differentially expressed *NtPP2C* genes were divided into 12 subfamilies (A-L) and an independent branch that does not belong to any subfamily. Among them, subfamily A contained the largest number of members, with 7 *NtPP2C* genes, followed by subfamily H with 4 *NtPP2C* genes. Transcript32471, which was significantly up-regulated in the ABA signaling pathway, was clustered in subfamily A, with the corresponding node receiving 94 bootstrap support (**Figure 6**). It is speculated that Transcript32471 may participate in the regulation of ABA-mediated stress responses in *N. tangutorum*.

**Figure 6:**
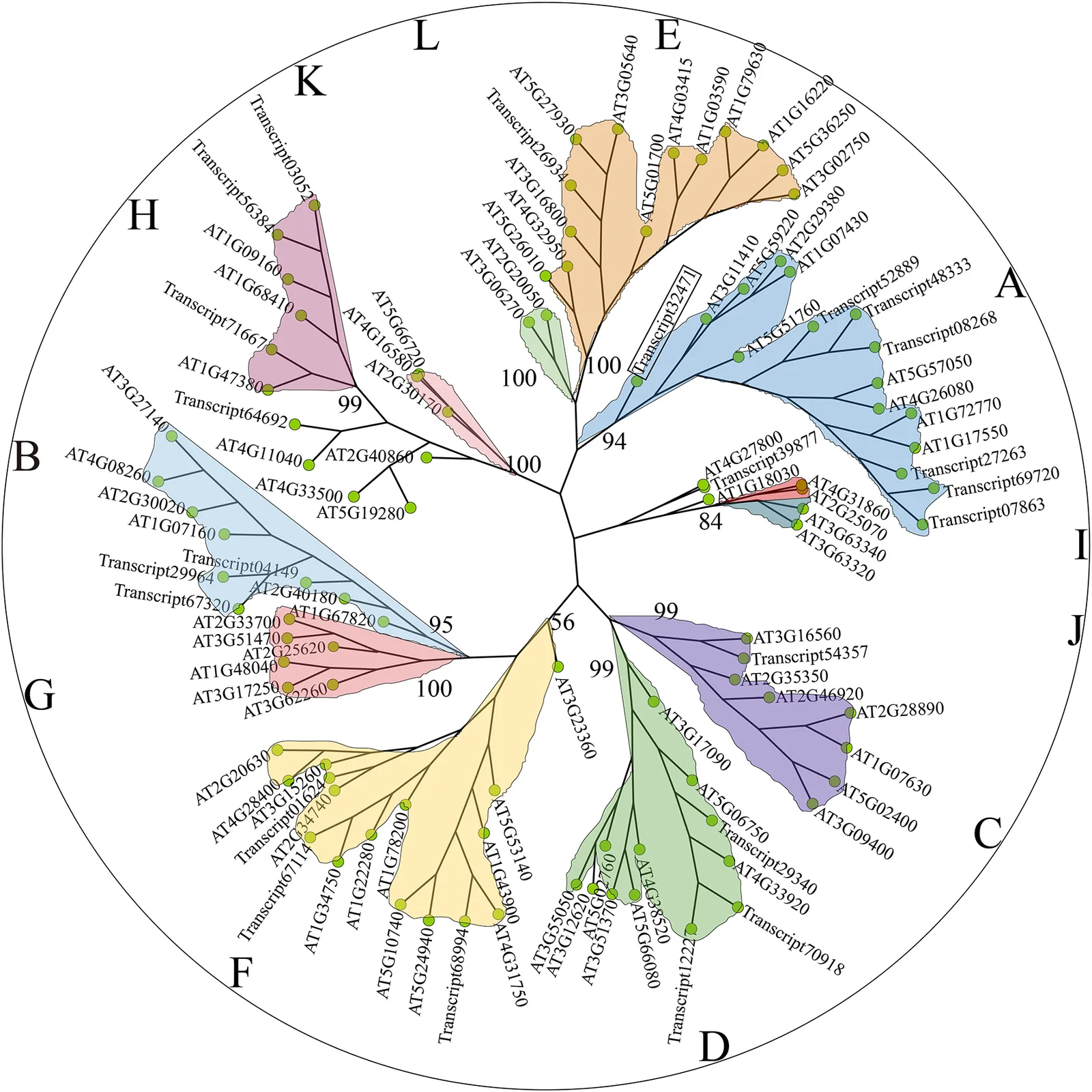
Phylogenetic tree of the *PP2C* gene family in *N. tangutorum* and *Arabidopsis thaliana*. Numbers at branch nodes are bootstrap support values (bootstrap percentages), reflecting the reliability of each branch. Letters **(A–L)** denote different subfamilies.

### 3.7 Quantitative real-time PCR verification

To verify the reliability of the transcriptome results, 12 DEGs, including 9 up-regulated genes and 3 down-regulated genes, were selected for qRT-PCR validation. As shown in **Figure 7**, the expression patterns of the 12 genes determined by qRT-PCR were generally consistent with those obtained from the transcriptome analysis, supporting the reliability of the RNA-seq data. Notably, the transcript abundance of Transcript32471 was significantly upregulated at 6 and 24 h after PEG treatment compared to the control (0 h). This consistent induction at both time points confirms its drought-responsive expression in *N. tangutorum* roots.

**Figure 7:**
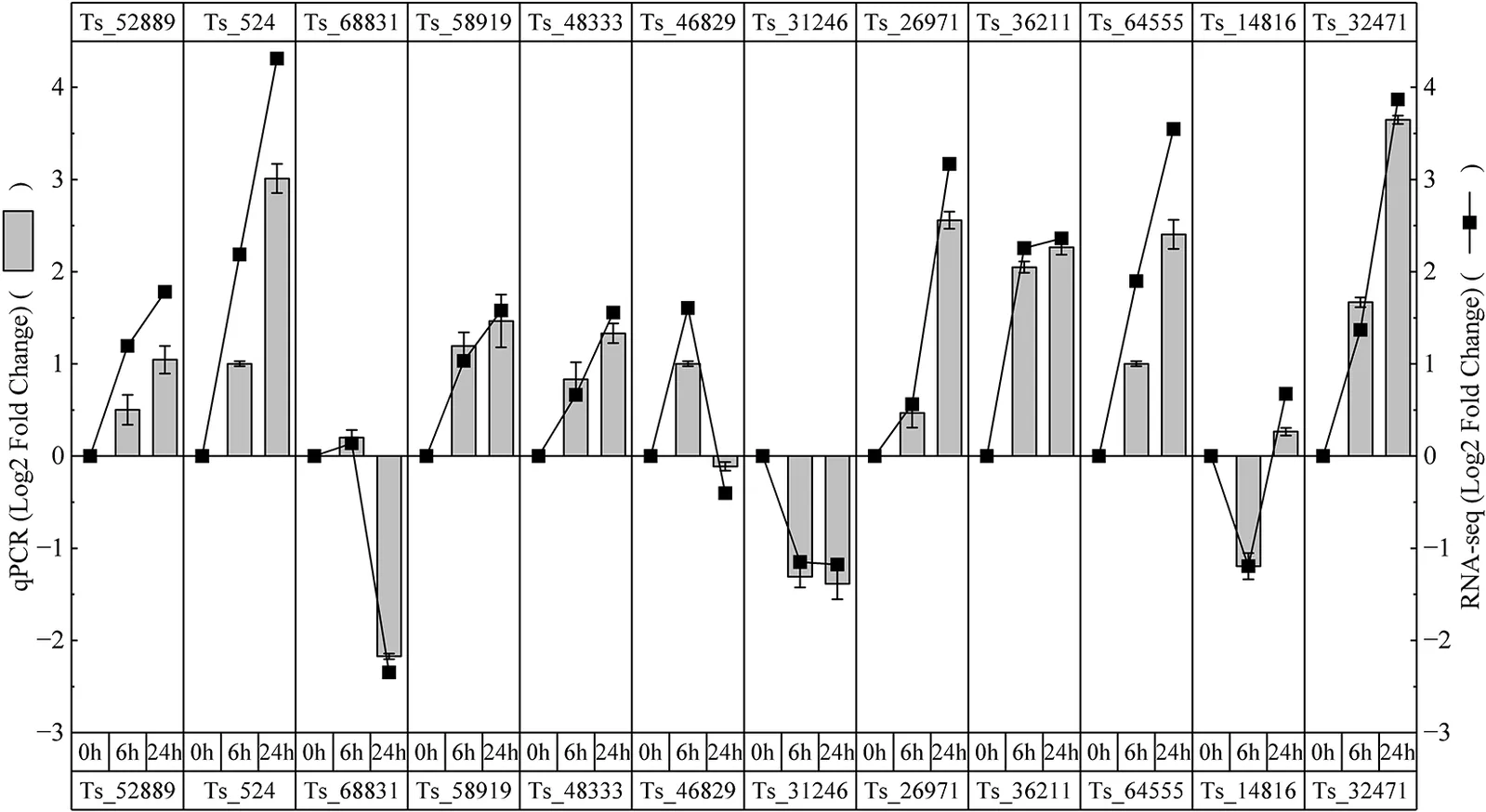
qRT-PCR validation of selected DEGs identified from the transcriptome analysis under PEG-induced drought stress. Data are presented as mean ± SE (n = 3 biological replicates).

### 3.8 Cloning and sequence analysis of *NtPP2C51-like*

To further elucidate the role of the drought-resistant key gene Transcript32471 in the drought response of *N. tangutorum*, we conducted functional verification of this gene. Using the cDNA of *N. tangutorum* as a template, PCR amplification was performed using primers specific to the target gene. The amplification product was recovered and purified, then ligated into the double-digested linearized vector *pART-CAM-FLAG*, followed by transformation into *E. coli* DH5α. Positive monoclonal colonies were selected for sequencing to obtain the complete sequence encoding the target gene. Sequence analysis revealed that the full length of target gene is 933 bp, encoding 296 amino acids (**Figure 8A**). BLAST analysis against the NCBI database showed that the deduced amino acid sequence of Transcript32471 exhibited the highest similarity to *Vitis vinifera PP2C51* (accession no. XP_002266149.1), with a query coverage of 94%, a sequence identity of 44.37%, similarity values of 59% and an E-value of 3 × 10^−55^. The encoded protein contained the conserved PP2C catalytic domain (PP2Cc), confirming its membership in the PP2C family (**Figure 8B**). Based on the significant sequence similarity to PP2C51-related proteins and the presence of the conserved PP2C catalytic domain, Transcript32471 was provisionally designated *NtPP2C51-like*. Phylogenetic analysis was performed by incorporating well-characterized PP2C51 homologs proteins from multiple model and non-model plant species. The results revealed that *NtPP2C51-like* is most closely related to *Melia azedarach* and *Dipteronia sinensis* with a bootstrap support value of 82, and its independent clade suggests that this gene may have undergone adaptive evolution in response to extreme stress (**Figure 8C**).

**Figure 8:**
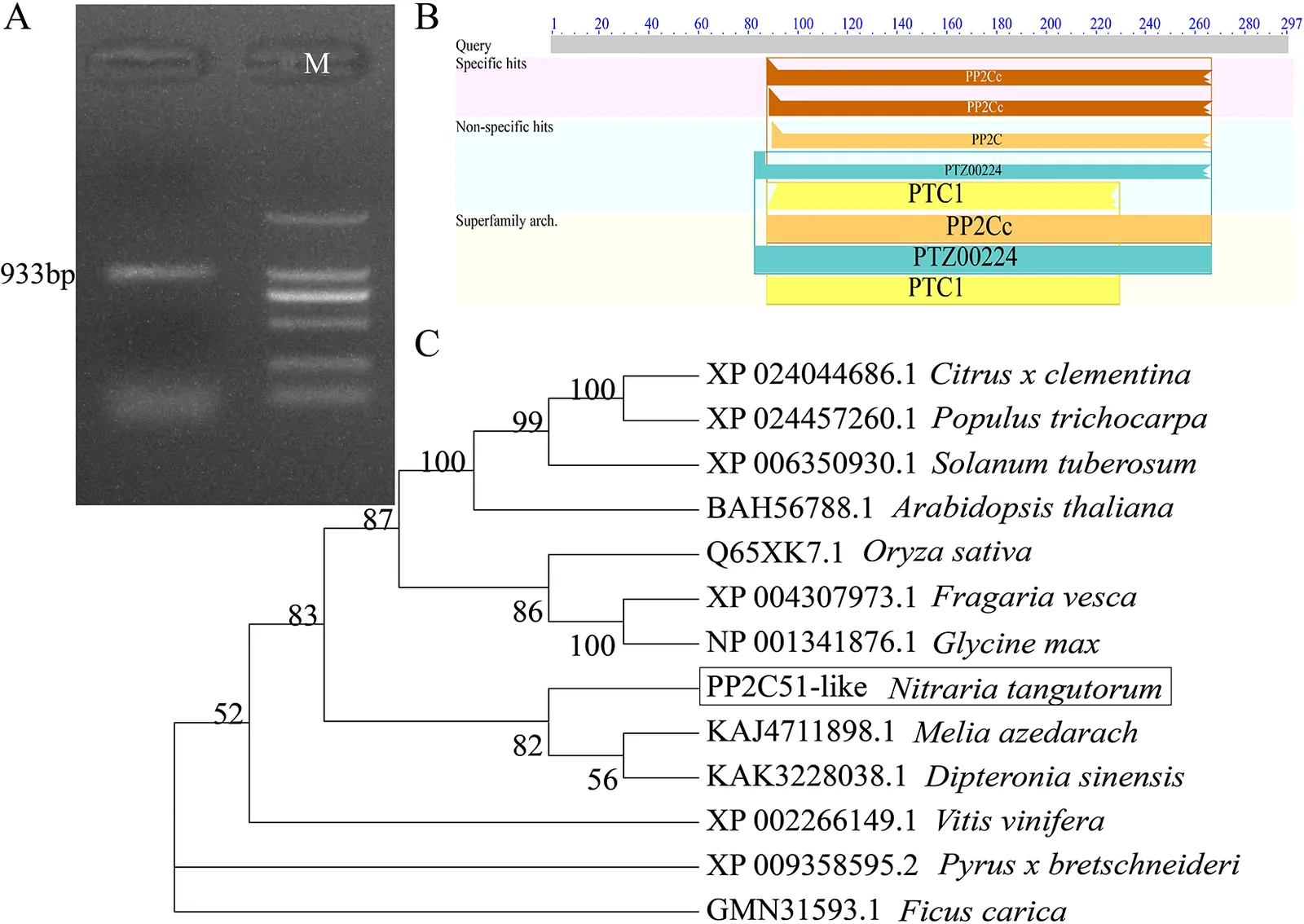
PCR products and sequence analysis of *NtPP2C51-like*. **(A)** PCR products of *NtPP2C51-like.* M: Marker DL2000; **(B)** Protein domain of *NtPP2C51-like*; **(C)** Phylogenetic tree of *NtPP2C51-like* homologous protein.

### 3.9 Identification of transgenic *N. benthamiana* lines and functional verification of *NtPP2C51-like*

The successfully sequenced recombinant plasmid *pART-CAM-FLAG-NtPP2C51-like* was introduced into *Agrobacterium* GV3101 competent cells using the freeze-thaw method. The recombinant expression vector *pART-CAM-FLAG-NtPP2C51-like* was used to transform *N. benthamiana* via *Agrobacterium*-mediated leaf disc transformation (**Figure 9A**). Genomic DNA was extracted from transgenic lines and identified by PCR using the specific primers 35S-F: AGAAGACGTTCCAACCACG and *NtPP2C51-like*-R: GGTTACCGCATCTTCCATCAC. Consequently, seven independent positive transgenic lines were successfully obtained (**Figure 9B**). As shown in **Figure 9C**, both wild-type and transgenic lines exhibited wilting under drought stress. However, compared with the wild-type, transgenic lines lost water more rapidly under drought stress, exhibited more severe wilting, and displayed weaker drought tolerance. The above results indicate that overexpression of *NtPP2C51-like* decreases drought resistance of plants. qRT-PCR was used to detect the expression of *NtPP2C51-like* gene in wild-type and transgenic *N. benthamiana* lines under drought stress at different time points (**Figure 9D**). The results showed that the transcript level of *NtPP2C51-like* in the transgenic lines remained relatively stable throughout the drought treatment period, with no substantial time-dependent changes, and were consistently significantly higher than those in the WT. The expression level in WT was highest after 5 days of drought stress, at which point the expression levels in OE1 and OE2 were 1.60- and 1.52-fold higher than that in the WT, respectively.

**Figure 9:**
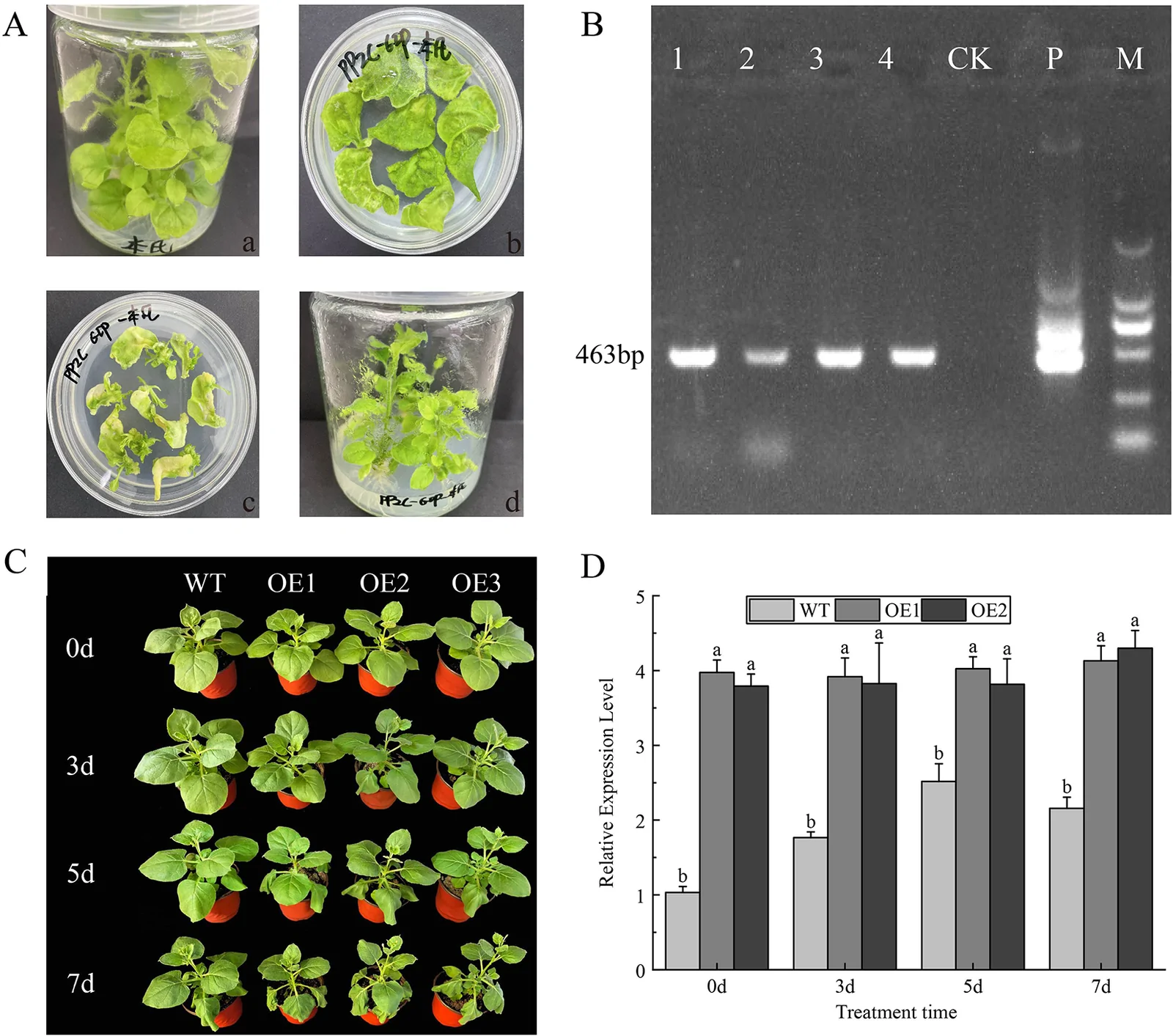
Identification of transgenic *N. benthamiana* lines and functional verification of *NtPP2C51-like*. **(A)** Genetic transformation of *NtPP2C51-like* in *N. benthamiana*. a: *N. benthamiana* tissue culture plantlets; b: Co-culture; c: Differentiation culture; d: Rooting culture; **(B)** PCR identification of transformed lines of *NtPP2C51-like*. M: Marker DL2000; 1-4: Transgenic *N. benthamiana* lines of *NtPP2C51-like*; CK: Blank control (ddH_2_O); P: Positive control (Plasmid); **(C)** Phenotypes of the wild type and *NtPP2C51-like*-transgenic *N. benthamiana* under drought stress. WT: wild type *N. benthamiana*; OE1, OE2, and OE3: Transgenic *N. benthamiana* lines; **(D)** Expression analysis of *NtPP2C51-like* genes in wild type and *NtPP2C51-like*-transgenic *N. benthamiana* under drought stress. Different lowercase letters above the error bars indicate statistically significant differences in expression level among the different lines under drought stress at same times (ANOVA, *p* < 0.05). Data are presented as mean ± SE (n = 3 biological replicates).

### 3.10 Effects of *NtPP2C51-like* overexpression on MDA and osmoprotectants in *N. benthamiana* under drought stress

To further explore the function of *NtPP2C51-like* in drought stress response, we measured and analyzed changes in physiological and biochemical indexes in wild-type and *NtPP2C51-like*-overexpressing *N. benthamiana* plants under drought stress at different time points. As shown in **Figure 10**, there was no significant difference in the contents of MDA, soluble sugar, soluble protein, and proline between wild-type and transgenic *N. benthamiana* under normal conditions. Under drought stress at different times, the contents of MDA, soluble sugar, soluble protein, and proline in both wild-type and transgenic *N. benthamiana* showed a continuous upward trend with the extension of stress time. The MDA content in transgenic *N. benthamiana* was significantly higher than that in the wild type under drought stress at different time points. The MDA content of OE1 and OE2 was 1.31–1.39-fold higher than that of wild type at 7 d of drought treatment. The contents of soluble sugar, soluble protein, and proline in transgenic *N. benthamiana* were significantly lower than those in the wild type under drought stress at different time points. At 7 d of drought treatment, the contents of soluble sugar, soluble protein, and proline in OE1 and OE2 were 57–61%, 87–90%, and 83–85%, respectively, of those in wild type.

**Figure 10:**
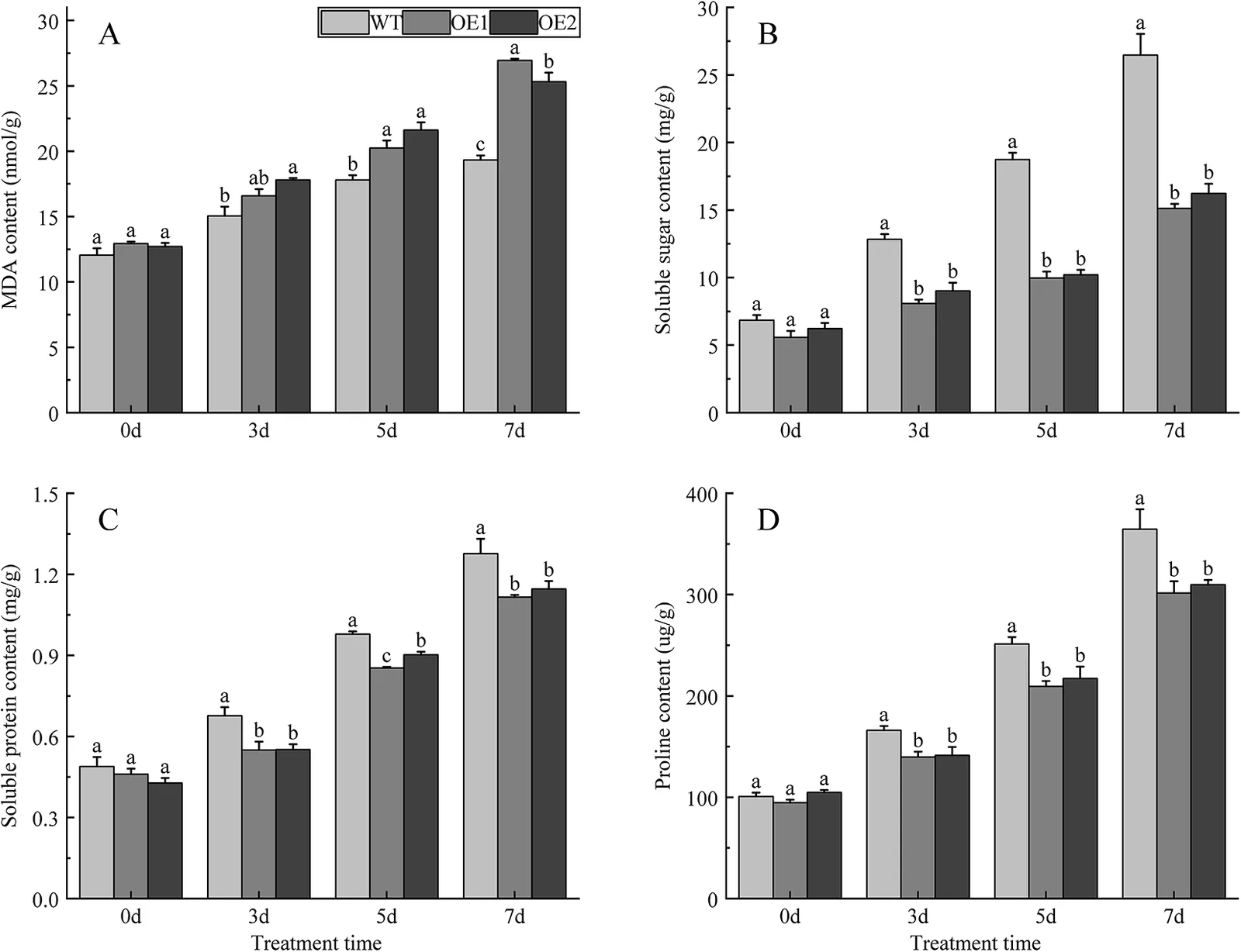
The changes in MDA and osmoprotectant contents in wild-type and *NtPP2C51-like* overexpressing *N. benthamiana* under drought stress. **(A)** MDA content; **(B)** Soluble sugar content; **(C)** Soluble protein content; **(D)** Proline content. 0 d, 3 d, 5 d, and 7 d represent different time points of drought stress treatment. Different lowercase letters above the error bars indicate statistically significant differences among different lines under drought stress at the same time. (ANOVA, *p* < 0.05). Data are presented as mean ± SE (n = 3 biological replicates).

### 3.11 Effects of *NtPP2C51-like* overexpression on starch and antioxidant enzyme activities in *N. benthamiana* under drought stress

As shown in **Figure 11**, there was no significant difference in starch content, POD activity, SOD activity, and CAT activity between wild-type and transgenic *N. benthamiana* under normal conditions. Under drought stress at different times, the starch content in transgenic and wild-type *N. benthamiana* decreased gradually with the extension of stress time, while the activities of POD, SOD, and CAT increased continuously with the prolongation of stress time. Under drought stress at different times, the starch content and the activities of POD, SOD, and CAT in transgenic *N. benthamiana* were significantly lower than those in wild-type plants. At 7 d of drought stress, the starch content and POD, SOD, and CAT activities in wild-type plants were 1.65–1.52-, 1.10–1.17-, 1.25–1.29-, and 1.17–1.14-fold, respectively, of those in OE1 and OE2.

**Figure 11:**
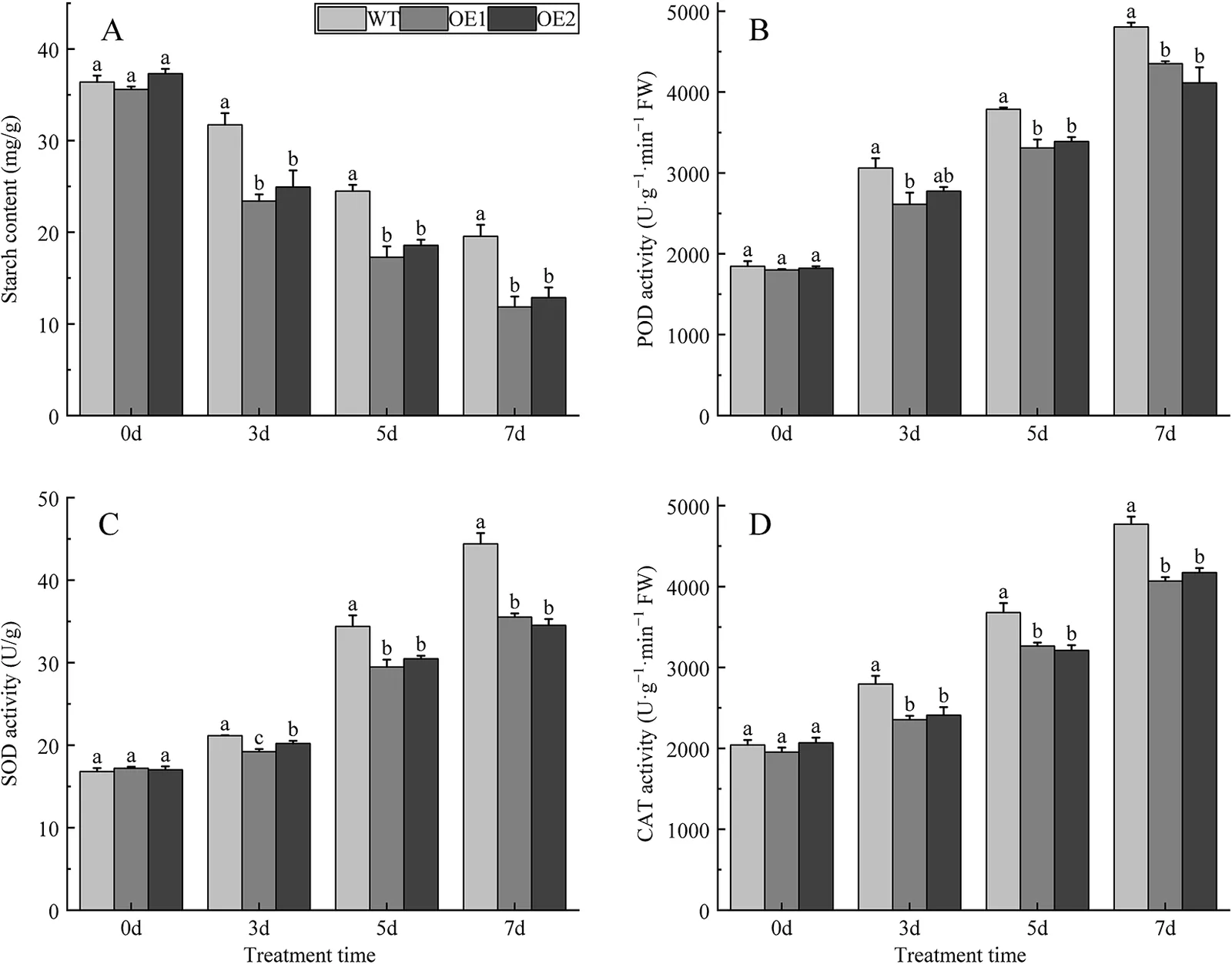
The changes in starch content and antioxidant enzyme activities in wild-type and *NtPP2C51-like* overexpressing *N. benthamiana* under drought stress. **(A)** Starch content; **(B)** POD activity; **(C)** SOD activity; **(D)** CAT activity. 0 d, 3 d, 5 d, and 7 d represent different time points of drought stress treatment. Different lowercase letters above the error bars indicate statistically significant differences among different lines under drought stress at the same time. (ANOVA, *p* < 0.05). Data are presented as mean ± SE (n = 3 biological replicates).

## 4 Discussion

Drought stress disrupts the water balance in plants, induces physiological and metabolic disorders, and hinders their normal growth and development (Haghpanah et al., 2024). Under drought stress, the original oxidative balance of plant cells is disrupted, thereby triggering oxidative stress and membrane lipid peroxidation in plants. As the end product of membrane lipid peroxidation, the content of MDA is directly proportional to the stress time (Zhou et al., 2022). In this study, the MDA content in roots, stems, and leaves of *N. tangutorum* seedlings increased continuously with the prolongation of stress time, which is consistent with the findings of Xiao et al., who found that the MDA content in the needles and roots of *Pinus massoniana* increased continuously with the passage of stress time (Xiao et al., 2021). Osmoregulation and the antioxidant defense system are the core mechanisms by which plants cope with drought stress (Yu et al., 2026). Plants maintain water potential balance and cell turgor stability by accumulating osmoregulatory substances such as soluble sugars, soluble proteins, and proline, thereby ensuring normal physiological and metabolic activities (Bandurska et al., 2017). This study measured the content of important osmoregulatory substances in roots, stems, and leaves of *N. tangutorum* seedlings under PEG-simulated drought stress at different durations. The results showed that the content of soluble sugar, soluble protein, and proline in different tissues of *N. tangutorum* seedlings increased with prolonged stress duration. The results are similar to those reported by Gao et al., who explored changes in osmoregulatory substance contents in three provenances of *N. tangutorum* under drought stress at different times (Gao et al., 2011). This study also revealed a specific organ allocation pattern, where soluble sugar content in leaves of *N. tangutorum* was significantly lower than that in roots and stems, while proline content was significantly higher in roots and stems under PEG-simulated drought stress at different times. This allocation pattern was also reflected in studies conducted by Franzisky et al. (2025) on *Phoenix dactylifera* and by Maraghni et al. (2014) on *Ziziphus lotus*. Starch is considered a decisive factor for plant to adapt to abiotic stress (Thalmann and Santelia, 2017). Evidence suggests that there is a linear inverse relationship between the severity of drought stress and starch content (Zhou et al., 2021). In this study, the starch content in roots, stems, and leaves of *N. tangutorum* seedlings decreased continuously with the passage of drought time. Antioxidant enzymes are the core components of the antioxidant defense system, which can effectively eliminate the accumulation of reactive oxygen species triggered by drought stress, alleviate membrane lipid peroxidation, and relieve oxidative damage to plant cells (Cheng et al., 2021). In this study, the activities of POD, SOD, and CAT in roots, stems, and leaves of *N. tangutorum* seedlings continued to increase with the prolongation of stress time. Notably, POD and SOD activities in roots were significantly lower than those in stems and leaves under PEG-simulated drought stress at different times. Similar findings were reported by Li et al. in their study on physiological and biochemical responses of alfalfa under drought stress (Li et al., 2007). This difference likely reflects tissue-specific divergence in antioxidant defense, as well as distinct physiological functions and ROS-generating environments across tissues. Leaves are directly involved in photosynthesis, and drought-induced disruption of photosynthetic electron transport can increase the risk of ROS accumulation, thereby requiring a stronger enzymatic antioxidant response. In addition to physiological and biochemical responses, plants also initiate a systematic drought-resistant regulatory mechanism at the molecular level by sensing stress signals and activating the expression of drought-related functional genes (Shi et al., 2025).

Transcriptome sequencing technology is a core means for analyzing the molecular mechanism of plant stress response (Zhou et al., 2024). Sabzeali et al. showed through transcriptome sequencing analysis that *Carthamus tinctorius* seedlings respond to drought stress by significantly upregulating related genes such as *BAM1*, *TPS6*, *CYP707A2*, and *PP2Cs* involved in beta-alanine biosynthesis, as well as ABA metabolism and signal transduction pathways (Sabzeali et al., 2026). This study conducted transcriptome sequencing on the roots of *N. tangutorum* seedlings under PEG-simulated drought stress at different time points, identifying a total of 2,455 differentially expressed genes across nine cDNA libraries. KEGG analysis showed that the differentially expressed genes were significantly enriched in plant hormone signal transduction and MAPK signal transduction pathways, which was consistent with the research results of Jiao et al. on the significant enrichment of differentially expressed genes in exploring the molecular mechanism of grape response to drought stress (Jiao et al., 2026). Signal transduction is a key link for plants to perceive environmental stimuli and initiate stress responses (Zhu, 2016). Plant hormone signal transduction, as a core component of the signal transduction network, plays a vital role in plant recognition, transmission and cascading amplification of drought signals, as well as mediating plant drought resistance responses (Qian et al., 2025). In this study, *N. tangutorum* seedlings responded to PEG-simulated drought stress by coordinating the signal transduction networks of auxin, cytokinin, gibberellin, abscisic acid, ethylene, and brassinolide, while upregulating key genes including *Aux/IAA*, *CRE1*, *AHP*, *GRD1*, *PP2C*, *EIN2*, and *BKI1*. Salehin et al. demonstrated in their study on drought resistance of *Arabidopsis thaliana* that Aux/IAA, a negative regulator in the auxin signaling pathway, mediates drought tolerance of *Arabidopsis thaliana* by modulating glucosinolate levels (Salehin et al., 2019). Tan et al. demonstrated in their exploration of the response mechanism of rapeseed seedlings to drought stress that rapeseed seedlings induce the upregulation of key genes *CRE1*, *AHP*, and *GRD1* in response to drought stress by coordinating plant hormone signaling pathways such as auxin, cytokinin, and gibberellin (Tan et al., 2024). Akter et al. also believe that genes related to hormone signaling pathways such as abscisic acid, brassinolide, and gibberellin are involved in the response of coconut to drought stress (Akter et al., 2025). The ABA signaling pathway serves as the core regulatory network for plants in response to drought stress, consisting of ABA receptors (PYR/PYL/RCAR), class A PP2C phosphatases, and SnRK2 kinases (Ali et al., 2020). Under drought stress, ABA accumulates abundantly and binds to its receptors PYR/PYL/RCAR, thereby inhibiting the activity of PP2C phosphatases, activating ABF transcription factors, initiating a series of drought-responsive gene expression, and ultimately enhancing plant tolerance to drought stress (Bhaskara et al., 2019). Meanwhile, the activated ABF can directly bind to the promoters of *PP2C* genes such as *ABI1* and *ABI2*, inducing the expression of *PP2C* genes, avoiding excessive activation of ABA signals, and ensuring the sustainability of plant stress responses (Fuchs et al., 2013; Jung et al., 2020).

Protein Phosphatase 2C (PP2C) is a class of serine/threonine protein phosphatases that strictly rely on Mg^2+^/Mn^2+^ activation, and its family members contain the conserved catalytic domain PP2Cc (Shen et al., 2024). As key signal regulatory factor, the PP2C protein family is widely distributed in higher plants (Xu et al., 2026). Fuchs et al. identified 80 PP2C members from *Arabidopsis thaliana*, classified them into 12 subfamilies (A-L) and 6 ungrouped branches, and confirmed that subfamily A *PP2Cs* are key negative regulators of ABA signal transduction (Fuchs et al., 2013). This study conducted a phylogenetic analysis of 23 differentially expressed *PP2C* genes in the transcriptome data and classified them according to the classification system of Fuchs et al., to explore the function of the Transcript32471 gene in the drought resistance response of *N. tangutorum*. Phylogenetic analysis indicated that Transcript32471 clustered with members of the subfamily A *PP2Cs* from *Arabidopsis thaliana*, suggesting that Transcript32471 may be involved in ABA-related drought responses in *N. tangutorum*. This is consistent with the findings of Lin et al., who demonstrated through overexpression in rice and *Arabidopsis* that *PhePP2CA13* from the moso bamboo subfamily A negatively regulates the response of moso bamboo to drought stress (Lin et al., 2025). To verify the function of Transcript32471, we constructed a Transcript32471 overexpression vector and obtained transgenic *N. benthamiana* via genetic transformation. After sequencing monoclonal colonies of *Escherichia coli*, a target sequence with a length of 933 bp was obtained. BLAST alignment against NCBI databases showed that this sequence exhibited the highest similarity to the *Vitis vinifera PP2C51* gene, so it was designated *NtPP2C51-like*. Bioinformatics analysis indicates that *NtPP2C51-like* belongs to the *PP2C* family and has the highest homology with *Melia azedarach* and *Dipteronia sinensis*. Liu et al. demonstrated in their exploration of the low-temperature response mechanism in flower organs of Yuluxiang pear that *PP2C51* mediates hormone signal transduction to respond to low-temperature stress (Liu et al., 2025). Yang et al. also demonstrated that repression of the transcription of *PP2C51-1* and *PP2C51-2* release SnRK2.6 from inhibition, thereby activating the SnRK2.6-SLAC1 phosphorylation cascade, and consequently promoting stomatal closure in Shanxin poplar (*Populus davidiana* × *P. bolleana*) in response to drought stress (Yang et al., 2026). Previous studies suggested that *NtPP2C51-like* may be involved in the response of *N. tangutorum* to adverse stress. In this study, phenotypic observations combined with physiological and biochemical analyses were performed on wild-type and *NtPP2C51-like* transgenic *N. benthamiana* under drought stress. The results showed that transgenic lines displayed more severe drought damage, higher MDA content, lower osmotic regulatory substance content, and antioxidant enzyme activities than wild-type lines. These findings suggest that *NtPP2C51-like* overexpression increases drought sensitivity in the heterologous *N. benthamiana* system and may indicate a negative role of *NtPP2C51-like* in the drought response of *N. tangutorum*. Similar conclusions are also reflected in the study of Zhang et al. Zhang et al. analyzed transcriptome data of *Populus trichocarpa* under drought stress and found that *PtrPP2C-9*, a member of subfamily A *PP2Cs*, was significantly upregulated by drought. Overexpression of *PtrPP2C-9* conferred enhanced sensitivity to osmotic stress and reduced stress tolerance in transgenic plants compared with the wild type (Zhang et al., 2026).

## 5 Study limitations

This study has three main limitations. First, drought responses were examined using short-term hydroponic PEG-6000 treatment (≤24 h), which imposes an abrupt osmotic shock that differs from the gradual soil dehydration experienced by N. tangutorum in natural arid environments. Thus, the observed physiological and molecular responses may not fully recapitulate drought responses under field conditions. Second, the 0 h sample served as the sole baseline reference, and no time-matched non-stressed controls were collected at 6, 12, and 24 h. This design makes it impossible to fully separate PEG-induced drought responses from diurnal rhythm or short-term developmental fluctuations. Third, functional characterization of NtPP2C51-like relied on transcript-level overexpression without Western blot confirmation. Thus, its proposed role in water-loss regulation remains inferential. Finally, plant water status and stomatal traits were not directly measured.

## 6 Conclusion

1. *N.tangutorum* seedlings respond to PEG-simulated drought stress by accumulating osmoregulatory substances, including soluble sugar, soluble protein, and proline, and enhancing antioxidant enzyme activities, including POD, SOD, and CAT.
2. Root transcriptome sequencing at key time points revealed that the plant hormone signal transduction pathway, along with hormone-related genes such as *IAA*, *GID1*, *ETR*, *EBF1/2*, and *PP2C*, plays a pivotal role in the PEG-simulated drought stress response of *N. tangutorum*.
3. Heterologous overexpression of *NtPP2C51-like* in *N. benthamiana* resulted in accelerated water loss and compromised osmotic adjustment and antioxidant defense under drought stress. These findings suggest that *NtPP2C51-like* may negatively regulate drought tolerance in the heterologous tobacco system, while its specific role in the drought response of *N. tangutorum* requires further validation.

## Data Availability

The raw transcriptome sequencing data generated in this study have been deposited in the NCBI Sequence Read Archive (SRA) under BioProject accession number PRJNA1469439, with nine corresponding SRA records available for the individual RNA-seq samples.
