# Supplementary material for: Regulatory mechanisms of Nitraria tangutorum in response to drought stress and functional verification of NtPP2C51-like
Source: Front Plant Sci. 2026 Oct 28;17:1964307. doi: 10.3389/fpls.2026.1964307 (PMC13751501; doi:10.3389/fpls.2026.1964307)
Supplement: Supplementary file 1 [file Table1.docx]

| Gene | Forward primer(5'-3') | Reverse primer(5'-3') | Purpose |
| --- | --- | --- | --- |
| *Ts_52889* | ATGACAAGGGCGGATGAGG | CCGACACCGTCTTAGAAGGC | Transcriptome validation |
| *Ts__524* | TGCCGATAATGGTTTCCCTA | GGCGATCAAGAAGTCACTCACT |  |
| *Ts__64555* | TCAAGATTGGCAGTTGGCAT | TGGCAAGAAGTAGAACCCGATT |  |
| *Ts__46829* | TGGCAAGGGACATAGTCAACA | CTGAAGGGCTCAAGGGATACA |  |
| *Ts__31246* | GCAACCACCACCAAAACGC | CGCCTTGATGGACTGAACGA |  |
| *Ts__32471* | CTAACTGCGGCAACTCAAGG | TACGATGCGGCTTCCACTC |  |
| *Ts__26971* | TGTGGGTGTAGTGGAAATAGCC | CAACTCGCCAAGGAAAGGA |  |
| *Ts__68831* | TTATGAAGGGGAGCACAACCA | TCGAGCAGGGGACTGAACTG |  |
| *Ts__14816* | ATGAACGGAGTTGATGCGG | CACCTGAGTGTAATGCCCACA |  |
| *Ts__58919* | GTGGTTCAGAATAATGGGGTGC | ACGACGGCTGTTGCTTGCT |  |
| *Ts__48333* | GGGGAGGCAGCATTTTCA | CCTGGAGTCACCGCAGTTAG |  |
| *Ts__36211* | AACAAGAAAACAGGGGCGAAC | ACCCATTGGGATCCAGCTC |  |
| *EF1-α* | ACGCCTTGCTTGCTTTCAC | GATTTCATCGTACCTTGCCTTT | Reference gene |
| *Ts__32471* | tcatttggagaggacacgcATGGATGGTCTGCAAATACCT | tcgtcatccttgtagtcgaaTTTGTTCCTTAAATAATTTAAAAAGACGGGG | Gene cloning |
| *NtPP2C51-like* | CCAACTGCGGCAACTCAA | GTCTCCTTCTCGTCGGTCCTAT | Expression analysis |
| *EF1-α* | CAAACTAAAGGCTGAGCGTGA | CCAGGACGATCAATCACAGTG | Reference gene |

Table S1 Primer sequences used in this study
